# Effect of regular resistance exercise, vitamin D, and calcium supplements on the bone mineral content and density in postmenopausal model of rats: An experimental study

**DOI:** 10.18502/ijrm.v19i1.8181

**Published:** 2021-01-25

**Authors:** Homa Hajisadeghi, Mohammad Ali Azarbayjani, Mohammadreza Vafaeenasab, Maghsoud Peeri, Mohamad Mahdi Modares Mosala

**Affiliations:** ^1^Department of Exercise Physiology, Central Tehran Branch, Islamic Azad University, Tehran, Iran.; ^2^Yazd Cardiovascular Research Center, Shahid Sadoughi University of Medical Sciences, Yazd, Iran.; ^3^Department of Nuclear Medicine, Baqiyatallah University, Tehran, Iran.

**Keywords:** Menopause, Vitamin D, Exercise, Calcium, Bone mineral density.

## Abstract

**Background:**

Postmenopausal osteoporosis progressively occurs due to alteration in the estrogen level during the menopause period, and subsequently elevates the risk of fractures.

**Objective:**

To evaluate the effect of regular resistance exercise, vitamin D, and calcium supplements on bone mineral content and density, postmenopausal rats used.

**Materials and Methods:**

In this experimental study, 72 female Sprague-Dawley rats (8-10 wk: 250 ± 15 gr) were ovariectomized and randomly divided into nine groups (n = 8/each): control, placebo, exercise (EX), exercise with vitamin D supplement (EX + D), exercise with calcium (EX + Ca), exercise with calcium and vitamin D (EX + Ca + D), vitamin D administration (D), calcium administration (Ca), and calcium and vitamin D (Ca + D) groups. Finally, the tail, hip, and lumbar bone mineral content, bone mineral density, bone thickness, and bone cells were evaluated in each group.

**Results:**

The tail, hip, and lumbar bone mineral density was increased significantly in the EX + Vit D group compared to the control group (p = 0.004, p = 0.007, p = 0.003, respectively). However, there were no significant changes in the bone mineral content of the hips and lumbar among the groups. Besides, bone thickness in the Ex + Vit D group was more than the other groups (p = 0.02). The number of osteoclast cells were decreased in the Ca + Vit D, Ex + Ca, Ex + Vit D, and Ex + Vit D + Ca groups compared to the control group. Osteocyte numbers were increased only in the Ex + Vit D group.

**Conclusion:**

Resistance exercise in combination with vitamin D and calcium have a positive effect on the bone mineral density and bone mineral content and might be able to prevent or delay the osteoporosis among elderly women. However, additional researches are needed to assess the molecular pathways of this process.

## 1. Introduction

Menopause is an inevitable phenomenon of the general aging in female's reproductive system (1) and is defined as the permanent termination of menstrual periods that occurs after the loss of ovarian follicle development (2). Although, the average menopausal age onset is about 51 yr, it can vary between 40 to 60 yr (3). Additionally, early menopause occurs in 1% of young women before 40 yr (4). Moreover, the menopausal estrogen loss leads to an accelerated bone loss and osteoporosis (5) that leads to reduction of bone mineral density (BMD) and subsequently the increase in osteoporotic fractures (6). Bone is defined as a mineralized connective tissue that includes four types of cells: osteoblasts, bone lining cells, osteocytes, and osteoclasts. Bone is responsible for several essential functions in the body, such as movement, support and protection of soft tissues, and calcium and phosphate storage. Despite its passive appearance, bone is an extremely dynamic organ that is continuously resorbed by osteoclasts and transformed by osteoblasts (7).

The essential role of vitamin D and its metabolites on bone absorption and formation has been identified for a long time (8). Vitamin D plays the main role in regulating bone cell proliferation and maturation, as well as bone mineralization and resorption (9). Moreover, severe vitamin D deficiency has been reported to lead to osteomalacia in adults (10). Therefore, deficiency in vitamin D can elevate the rate of bone turnover and bone loss by increasing bone resorption in postmenopausal women (11). Some studies have demonstrated that Vitamin D receptors (VDRs) are expressed in reproductive systems including ovaries, endometrium, and placenta (12, 13). Furthermore, several studies have reported the role of vitamin D + calcium supplements in maturation of the ovarian follicles (13, 14). Additionally, the efficacy of calcium intake together with Vitamin D supplement has been demonstrated as an essential intervention for preventing osteoporosis due to postmenopausal conditions (15) by increasing BMD (16). Despite the genetic efficacy on the age of menopause, it is theoretical that lifestyle factors such as diet and physical activity play a significant role in ovarian age (17). Previous studies confirmed that physical activity and, especially, physical exercise are effective in the reduction of clinical fracture in postmenopausal women (18, 19). Muir and colleagues in 2013 published a study to assess the effect of physical activity on bone density in postmenopausal woman. Their results show that, overall, a regular exercise was effective for bone density in postmenopausal women (20). In addition, several previous studies have shown that physical activity, particularly, regular exercise program is the main way to maintain the BMC and prevent bone loss in women (21, 22).

Thus, it is an interesting question - whether an exercise program in combination with calcium and vitamin D supplements can play a supporting role in the maintenance or strengthening of bone mineral content (BMC) and BMD post menopause. Accordingly, the aim of this study was to evaluate the efficacy of regular exercises in combination with vitamin D and calcium supplements on the BMD in postmenopausal rat model.

## 2. Materials and Methods

### Animals and ethics

In this experimental study, 72 Sprague-Dawley rats, aged eight months with an initial weights of approximately 250 ± 15 gr were used. They the rats were assigned randomly into nine groups:

• Control group: underwent ovariectomy and were kept in cages for 4 months without any type of exercise.

• Ovariectomy calcium supplement group (Ca): underwent ovariectomy and after two months of housing, animals were administered 35 mg/kg calcium supplement (Calcium, Arian Salamat, Iran) by oral gavage for two months.

• Ovariectomy vitamin D supplement group (Vit D): underwent ovariectomy and after two months of housing, animals were injected 10,000 IU/wk vitamin D supplement (Vitamin D3 1000 IU, Health Aid, England) for two months.

• Ovariectomy vitamin calcium and D supplement group (Ca + Vit D): underwent ovariectomy and after two months of housing, animals were administered 35 mg/kg calcium and 10,000 IU/week vitamin D supplements for two months.

• Ovariectomy exercise group (Ex): underwent ovariectomy and performed two months of resistance training that started at the same time as the intact group.

• Ovariectomy exercise and vitamin D supplement group (Ex-Vit D): underwent ovariectomy and performed two months of resistance training. Animals were injected 10,000 IU/week vitamin D supplement that started at the same time as the intact group.

• Ovariectomy exercise and vitamin Ca supplement group (Ex-Ca): underwent ovariectomy and performed two months of resistance training. Animals were administered 35 mg/kg calcium supplement by oral gavage for two months.

• Ovariectomy exercise and calcium and vitamin D supplement group (Ex-Ca & Vit D): underwent ovariectomy and performed two months of resistance training. Animals were administered 35 mg/kg calcium and 10,000 IU/wk vitamin D supplement for two months after ovariectomy.

• Placebo group: animals were only administrated sesame oil for two months after ovariectomy.

### Ovariectomy surgery protocol

For ovariectomy operation, all animals were anesthetized intraperitoneally with a ketamine-xylazine mixture (61.5-7.6 mg/kg), and subsequently, ovaries were removed by a bilateral incision through the skin and completely excised from a dorsal approach (23). The site was sutured and all animals recovered two months after the surgical procedures.

### Resistance training protocol

For Resistance training, a 1-m ladder with 2-cm grid ladder inclined at 85° was used. Prior to the resistance training, rats underwent a familiarization week with a ladder and climbing. Weights attached to their tails and the initial weight was 30% of their body weight, which was increased to 100% of their body weight gradually throughout the eight weeks. Weights were fastened to the upper portion of the tail. The resistance training included three sets of five repetitions with a 30-sec rest interval between the reps and 3 min between the sets (24).

### Measurement methods

Animals were sacrificed by decapitation after the last resistance exercise sessions and after the supplement administration. Whole body was fixed in formaldehyde 10% to measure BMD (mg/cm${}^{2}$) and BMC (mg) of the total body, tail, hips, lumbar, and femur using dual-energy X-ray absorptiometry (387A030, Norland company, USA) (25). Femoral bone weight was measured using a digital scale (Taishi, China). Soon after, femur was retired and decalcified in hydrochloric acid/formic acid for 24 hr. Next, the samples were briefly rinsed in water and transferred to ammonia solution to neutralize acids and left for 30 min. Femurs were dehydrated in a tissue processor. After dehydration in ethanol series, the samples were embedded in paraffin and 5-µm sections were made. For histological evaluation, a standard length of 2 mm of each section of each bone was obtained to analyze the number of osteoblast, osteoclast, and osteocyte cells using a light microscope (Olympus BX51, Japan) with hematoxylin-eosin staining. Measurements were made on digitized images using the digimizer software. The total number of osteoblast, osteoclast, and osteocyte cells per square millimeter was calculated. Also, a cross-sectional area of bone tissue was used to measure the bone body thickness, bone lumen diameter, and bone total diameter. The mean bone lumen diameter (LD) and total diameter (TD) were derived by taking the average of two diameters, D1 and D2, at right angles. For each slide, the mean of 10 random fields was selected for measurements (26).

### Ethical considerations

Animals had free access to water and food and at a controlled temperature of 22 ± 2°C under 12-hr light/dark cycles. This study was approved by the Committee of Yazd Reproduction Sciences Institute, Shahid Sadoughi University of Medical Sciences, Yazd, Iran (IR.SSU.RSI.REC.1398.020) and all protocols were performed according to the National Institute of Health Guidelines for the Care and Use of Laboratory Animals (NIH Publications No. 8023, revised 1978).

### Statistical analysis

Data were analyzed using the SPSS software, version 20 (SPSS Inc., USA) and expressed as means ± SD. The statistical analysis was done initially by a one-way analysis of variance. A significance level of p < 0.05 and p < 0.001 was used for all comparisons.

## 3. Results

**Figure 1:**
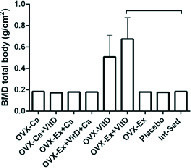
Bone mineral density was significantly increased in the Ex-Vit D group compared to the control group (p ≤ 0.001). The statistical analysis was done initially by a one-way analysis of variance. BMD: Bone mineral density; Ex: Resistant exercise; Ca: Calcium supplement; Vit D: Vitamin D supplement.

**Figure 2:**
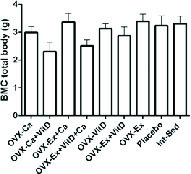
There was no significant differences in the bone mineral content of rats among the different groups. The statistical analysis was done initially by a one-way analysis of variance. BMC: Bone mineral content; Ex: Resistant exercise; Ca: Calcium supplement; Vit D: Vitamin D supplement.

Resistant exercise with Vit D administration in ovariectomized rats significantly increased tail, hip, and lumbar BMD compared to the control group, p = 0.004, p = 0.007, p = 0.003, respectively (Table I). Also, there was an increasing trend in the femur BMD of the Ex + Vit D group in comparison with the control group (p = 0.009, Table I). The total BMD showed an increase in the Ex + Vit D group over the control group (Figure 1). However, higher tail and femur BMC were observed in the Ex + Vit D group compared to the control group (p = 0.005 and p = 0.002, respectively. No significant changes were observed in the hips and lumbar BMC compared to the control group (Table II). There was no statistically significant difference in the total BMC comparison with the control group (Figure 2).

**Figure 3:**
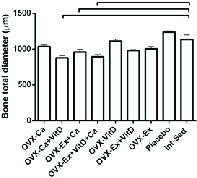
Data shows that the total bone diameter was significantly decreased in the Ca + Vit D, Ex + Ca, Ex + Vit D + Ca groups compared to the control group (p ≤ 0.001). The statistical analysis was done initially by a one-way analysis of variance. Ex: Resistant exercise; Ca: Calcium supplement; Vit D: Vitamin D supplement.

**Figure 4:**
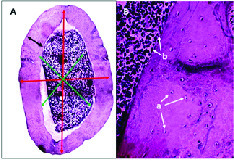
(A) Cross-section of femur was used to measure lumen diameter (green arrow), bone thickness (black arrow), and total bone diameter (red arrow) (Magnification 40×). (B) Number of osteocyte (a) and osteoblast (b) cells in femoral bone (Magnification 400×).

Moreover, no significant changes were observed between the groups in femoral bone weight (Table III). In addition, reduction of lumen diameter was indicated in groups that were administrated Ca supplement during the study compared to the control group. However, lumen diameter was lower in the Ex + Vit D group than the control group; a significant decrease was seen in the lumen diameter of the Ca + Vit D, Ex + Ca, and Ex + Vit D + Ca groups. Interestingle, bone thickness in the Ex + Vit D group was more than the control groups (p = 0.02; Table III). In addition, the total bone diameter was significantly decreased in the Ca + Vit D (p = 0.000), Ex + Ca (p = 0.018), and Ex + Vit D + Ca (p = 0.001) groups compared with the control group (Figure 3 and 4A).

Figure 4B shows osteocyte and osteoblast cells in the cross-section of the femoral bone. Also, there were no significant differences in the number of osteoblast cells among the control group (Table IV). However, the number of osteoclast cells were decreased in the Ca + Vit D, Ex + Ca, Ex + Vit D, and Ex + Vit D + Ca groups compared to the control group; osteocyte numbers were increased only in the Ex + Vit D group.

**Table 1:** Effect of resistant exercise and administration of calcium 0. and vitamin D supplements on ovariectomized rat on bone mineral density of tail, hips, lumbar, and femur


**Group**	**BMD tail (mg/cm${}^{2}$)**	**BMD hips (mg/cm${}^{2}$)**	**BMD lumbar (mg/cm${}^{2}$)**	**BMD femur (mg/cm${}^{2}$)**
**Ca**	0.19 ± 0.013	0.18 ± 0.009	0.19 ± 0.006	0.17 ± 0.004
**Ca + Vit D**	0.19 ± 0.006	0.18 ± 0.006	0.19 ± 0.003	0.18 ± 0.014
**Ex + Ca **	0.17 ± 0.013	0.30 ± 0.128	0.18 ± 0.007	0.16 ± 0.004
**Ex + Vit D + Ca **	0.20 ± 0.006	0.17 ± 0.003	0.18 ± 0.005	0.17 ± 0.006
**Vit D**	0.19 ± 0.009	0.18 ± 0.007	0.19 ± 0.009	0.18 ± 0.010
**Ex + Vit D**	0.62 ± 0.266*	0.52 ± 0.215*	0.48 ± 0.193*	0.40 ± 0.142*
**Ex**	0.89 ± 0.298	0.68 ± 0.202	0.69 ± 0.206	0.65 ± 0.202
**Placebo**	0.20 ± 0.010	0.18 ± 0.007	0.18 ± 0.006	0.16 ± 0.004
**Control **	0.19 ± 0.002	0.15 ± 0.01	0.20 ± 0.014	0.16 ± 0.005
**P-value**	0.004	0.007	0.003	0.009
Data are expressed as Mean ± SD. *p ≤ 0.05 and **p ≤ 0.001 when compared to the control group and the statistical analysis was done initially by a one-way analysis of variance. BMD: Bone mineral density; Ex: Resistant exercise; Ca: Calcium supplement; Vit D: Vitamin D supplement				

**Table 2:** Effect of resistant exercise and administration of calcium and vitamin D supplements on ovariectomized rat on bone mineral content of tail, hips, lumbar, and femur


**Group**	**BMC tail (mg)**	**BMC hips (mg)**	**BMC lumbar (mg)**	**BMC femur (mg)**
**Ca**	0.16 ± 0.039	0.58 ± 0.102	0.62 ± 0.076	0.39 ± 0.070
**Ca + Vit D**	0.18 ± 0.018	0.42 ± 0.046	0.52 ± 0.110	0.35 ± 0.043
**Ex + Ca **	0.26 ± 0.01	0.58 ± 0.051	0.65 ± 0.083	0.40 ± 0.047
**Ex + Vit D + Ca **	0.24 ± 0.039	0.35 ± 0.0267	0.47 ± 0.106	0.34 ± 0.035
**Vit D**	2.50 ± 0.900	2.29 ± 1.210	0.89 ± 0.344	0.39 ± 0.144
**Ex + Vit D**	3.4 ± 1.334**	2.70 ± 0.981	1.17 ± 0.261	2.75 ± 1.009**
**Ex**	0.26 ± 0.050	0.61 ± 0.087	0.69 ± 0.099	0.41 ± 0.043
**Placebo**	1.06 ± 0.043	0.34 ± 0.069	0.26 ± 0.057	0.32 ± 0.053
**Control**	0.27 ± 0.042	0.66 ± 0.097	0.816 ± 0.077	0.53 ± 0.042
**P-value**	0.005	0.016	0.023	0.002
Data are expressed as Mean ± SD. **p ≤ 0.001 when compared to the control group and the statistical analysis was done initially by a one-way analysis of variance. BMC: Bone mineral content; Ex: Resistant exercise; Ca: Calcium supplement; Vit D: Vitamin D supplement				

**Table 3:** Effect of resistant exercise and administration of calcium and vitamin D supplements on ovariectomized rat on the femoral bone weight, lumen diameter, and bone thickness


**Group**	**Femoral bone weight (gr)**	**Lumen diameter**	**Bone thickness**
**Ca**	1.85 ± 0.160	747.75 ± 26.771	287.94 ± 13.937
**Ca + Vit D**	1.92 ± 0.314	608.99 ± 12.261**	270.13 ± 22.444
**Ex + Ca **	1.98 ± 0.207	685.68 ± 38.158	272.04 ± 16.918
**Ex + Vit D + Ca **	1.71 ± 0.0916	658.44 ± 26.625**	234.85 ± 14.880
**Vit D**	1.50 ± 0.0885	819.58 ± 6.778	292.24 ± 24.276
**Ex + Vit D**	1.68 ± 0.198	598.24 ± 25.140**	381.89 ± 16.139**
**Ex**	1.61 ± 0.191	717.50 ± 41.979	286.51 ± 21.294
**Placebo**	1.85 ± 0.076	963.42 ± 23.024	273.16 ± 9.217
**Control**	2.07 ± 0.188	845.73 ± 56.764	287.70 ± 17.131
**P-value**	0.501	≤ 0.001	≤ 0.001
Data are expressed as Mean ± SD. **p ≤ 0.001 when compared to the control group and the statistical analysis was done initially by a one-way analysis of variance. Ex: Resistant exercise; Ca: Calcium supplement; Vit D: Vitamin D supplement			

**Table 4:** Effect of resistant exercise and administration of calcium and vitamin D supplements on ovariectomized rat on the number of osteoblast, osteoclast, and osteocyte cells


**Group**	**Osteoblast**	**Osteoclast**	**Osteocyte**
**Ca**	19.17 ± 1.61	2.00 ± 0.000**	45.00 ± 0.750
**Ca + Vit D**	16.75 ± 0.52	2.42 ± 0.220**	38.50 ± 0.947
**Ex + Ca **	19.25 ± 0.52	3.67 ± 0.167*	37.50 ± 0.764
**Ex + Vit D + Ca **	18.00 ± 1.18	2.33 ± 0.167**	41.83 ± 3.595
**Vit D**	16.92 ± 0.58	5.17 ± 0.547	31.58 ± 4.547
**Ex + Vit D**	18.08 ± 0.60	3.92 ± 0.333*	63.08 ± 6.325**
**Ex**	17.58 ± 0.58	3.58 ± 0.167**	44.47 ± 4.775
**Placebo**	17.92 ± 0.68	4.33 ± 0.220	33.25 ± 4.003
**Control **	17.83 ± 1.58	5.16 ± 0.083	34.75 ± 2.883
**P-value**	0.36	≤ 0.001	≤ 0.001
Data are expressed as Mean ± SD. *p ≤ 0.05 and **p ≤ 0.001 when compared to the control group and the statistical analysis was done initially by a one-way analysis of variance. Ex: Resistant exercise; Ca: Calcium supplement; Vit D: Vitamin D supplement			

## 4. Discussion

Osteoporosis is identified as a disorder affecting elderly people, especially postmenopausal women. Hence, the determination of a beneficial lifestyle for elderly women seems to be necessary in order to reduce the incidence of postmenopausal syndromes. In the current study, we assessed the effect of resistance exercise, vitamin D and calcium supplements on ovariectomized rats as a model of menopause. Our findings showed the resistance exercise in combination with vitamin D supplement had a positive effect on the maintenance of BMD and BMC in OVX rats. There was a significant difference in the BMD and BMC in Ex + Vit D group in comparison with the other groups. It was reported that the resistance exercise can lead to increasing BMD at the muscle site by enhancement of muscle size and strength (27). Also, the BMC and BMD have been reported to reveal an increase up to 20% in the loaded bone regions in athletes (28). In a recent study, Beavers evaluated the change in BMD during weight loss with various physical activity including resistance and aerobic exercise in older adults. Their result showed that the resistance exercise was osteo-protective in comparison with the caloric restriction in elderly women (29).

According to result of Khalil study in 2015, vitamin D supplement reduced the bone resorption marker in elderly women (9). Several studies reported the important role of vitamin D to prevent the decrease in the bone density and the risk of bone fracture among elderly women (30, 31). Additionally, it was mentioned that some analogs of vitamin D such as 2-Methylene-19-nor (20S)-1α25-dihydroxyvitamin D3 (2MD) contribute in the enhancement of bone mass and are supposed to be a promising treatment for osteoporosis in postmenopausal women (30). Furthermore, vitamin D contributes in regulating calcium homeostasis as a part of bone metabolism procedure that displays the importance of vitamin D presence in bone health (32). On the other hand, several investigations supposed that the dietary calcium intake or calcium supplements contribute in the improvement of BMC and BMD among postmenopausal women. Nonetheless, some others revealed that the calcium supplements alone probably cannot be adequate to decrease fracture risk and other parameters such as vitamin D is required (33). This findings were similar to our result.

Further, it has been established that exercise can increase BMD, BMC, and bone strength (33). Sport researcher believed that continuing pressure and tension to bones is a valuable factor to prevent osteoporosis (34). Although, the role of exercise alone was clear, future studies were needed to define optimal physical activity and additional supplements for preventing osteoporosis and bone fractures (35, 36). Exercise can elevate seroma calcium and vitamin D levels in body but this increase depends on the type, intensity, and time of exercise (37). Mason and colleagues in 2011 reported that vitamin D level can increase after exercise for low-weight program (38). In the current study, an increase in the BMD and BMC in the Ex + Vit D group was observed. These significant changes might be due to the elevation of vit D after exercise. On the other hand, in this group, the elevation in the vitamin D level create from two ways: (1) exogenous vitamin D by supplement intake and (2) endogenous vitamin D by exercise. So, this elevation might be responsible for BMD and BMC increase in this group in compared to other groups.

The bone thickness was morphologically compared between all study groups. There was a significant difference in the Ex + Vit D groups in comparison with the other groups. On the other hand, our finding can confirm that the combination of resistance exercise and vitamin D has a positive effect on the maintenance of bone thickness and prevention or delay of osteoporosis. In the present study, osteocytes were counted in all groups and a significant difference was observed in the Ex + Vit D group in comparison with the other groups that confirmed our previous findings and revealed the promising potential of resistance exercise together with vitamin D intake in reducing bone resorption by decreasing the rate of osteocytes apoptosis. Toumi and colleagues have illustrated that the physical activity alone does not significantly reduce the rate of osteocytes apoptosis in all sham and OVX groups (39). While it is reported that endurance exercise plays an important role in preservation of cancellous bone (40). Moreover, another study showed that exercise would be able to enhance osteogenesis in OVX rats (41). Abnormal proliferation and activity of osteoclasts lead to various bone disorders such as osteoporosis causing decreased BMD and elevate the possibility of bone fractures (7).

Besides, there was a significant increase in the number of osteocyte in the Ex + Vit D group in comparison with the other groups. Some studies have reported that exercise can elevate estrogen level in mice serum, and this elevation can prevent osteoclast activity and bone resorption (42, 43). Some researchers revealed that vitamin D level in serum can be raised in animals who were in exercise program (38, 44). In this issue, exercise might have led to the increase in the VDRs, and upsurge in the production of active vitamin D. Finally, this factor perhaps can affect osteoblasts activity and prevent osteoporosis. We also found that the osteoclast was decreased in the Ca + Vit D, Ca + Ex, and Ex + Vit D groups. In addition, our result showed that vitamin D supplementation alone may not be sufficient for the reduction of osteoclasts and decrease in bone cell apoptosis; however, the effective level of Ca requires deactivation of the osteoclasts.

## 5. Conclusion

Our findings demonstrated that resistance exercise in combination with vitamin D intake represents effective preventive and therapeutic strategies able to prevent or delay the onset of osteoporosis. However, additional investigations are required for the evaluation of all effective parameters on bone health, particularly from the molecular point of view.

##  Conflict of Interest

The authors declare that there is no conflict of interest.
